# Bispecific antibodies in multiple myeloma treatment: A journey in progress

**DOI:** 10.3389/fonc.2022.1032775

**Published:** 2022-10-18

**Authors:** Shih-Feng Cho, Tsung-Jang Yeh, Kenneth C. Anderson, Yu-Tzu Tai

**Affiliations:** ^1^ Division of Hematology & Oncology, Department of Internal Medicine, Kaohsiung Medical University Hospital, Kaohsiung Medical University, Kaohsiung, Taiwan; ^2^ Faculty of Medicine, College of Medicine, Kaohsiung Medical University, Kaohsiung, Taiwan; ^3^ Center for Cancer Research, Kaohsiung Medical University, Kaohsiung, Taiwan; ^4^ LeBow Institute for Myeloma Therapeutics and Jerome Lipper Multiple Myeloma Center, Dana-Farber Cancer Institute, Harvard Medical School, Boston, MA, United States

**Keywords:** multiple myeloma, immunotherapy, bispecific antibody, bispecific T-cell engager, BCMA, GPRC5D, FcRH5, CD38

## Abstract

The incorporation of novel agents and monoclonal antibody-based therapies into the treatment of multiple myeloma (MM) has significantly improved long-term patient survival. However, the disease is still largely incurable, with high-risk patients suffering shorter survival times, partly due to weakened immune systems. Bispecific molecules, including bispecific antibodies (BisAbs) and bispecific T-cell engagers (BiTEs), encourage immune cells to lyse MM cells by simultaneously binding antigens on MM cells and immune effector cells, bringing those cells into close proximity. BisAbs that target B-cell maturation antigen (BCMA) and GPRC5D have shown impressive clinical activity, and the results of early-phase clinical trials targeting FcRH5 in patients with relapsed/refractory MM (RRMM) are also promising. Furthermore, the safety profile of these agents is favorable, including mainly low-grade cytokine release syndrome (CRS). These off-the-shelf bispecific molecules will likely become an essential part of the MM treatment paradigm. Here, we summarize and highlight various bispecific immunotherapies under development in MM treatment, as well as the utility of combining them with current standard-of-care treatments and new strategies. With the advancement of novel combination treatment approaches, these bispecific molecules may lead the way to a cure for MM.

## Introduction

The treatment landscape of multiple myeloma (MM), the second most common hematologic malignancy in America, has been revolutionized in recent decades by proteasome inhibitors (PI), including bortezomib, and immunomodulatory drugs (IMiDs), including thalidomide and lenalidomide (1–3). The second generation of these drugs, such as carfilzomib and ixazomib (PIs) and pomalidomide (IMiDs), further improved the response rate, survival and safety profile (4–6). Moreover, the incorporation of autologous stem cell transplantation in eligible patients has also resulted in better survival and more durable disease control (7, 8). However, MM remains an almost incurable disease for most patients, since treatment-resistant clones eventually emerge and evolve, leading to a low 5-year overall survival (OS) of about 50% (9). The clinical outcomes of patients with relapsed or refractory MM (RRMM) are dismally poor because of their diminishing physical performance and the gradually decreased durability of response with successive lines of anti-MM therapy (10, 11).

Immunotherapy has proven revolutionary in many cancer fields, yet progress has been slow in MM due to its generally immunosuppressive microenvironment, which impairs the efficacy of immunotherapy (12). The malignant MM cells closely interact with the surrounding bone marrow (BM) accessory cells, including BM stromal cells (BMSC) (13), osteoclasts (OC) (14, 15), regulatory T or B cells (Treg or Breg) (16–18), myeloid-derived suppressor cells (MDSC) (19), tumor-associated macrophages (TAMs) (20), and plasmacytoid dendritic cells (21). These cells promote the growth and chemoresistance of MM cells by inducing or secreting cytokines such as interleukin-6 (IL-6) (22), interleukin-10 (IL-10) (23), transforming growth factor-beta (TGF-β) (24), a proliferation-inducing ligand (APRIL) (25), and heat shock proteins (26). These accessory cells and cytokines play key roles in promoting tumor immune escape, inhibiting tumor-specific T effector cells, inducing T-cell anergy, and increasing the number of Tregs, leading to an immunosuppressive microenvironment.

In recent years, immunotherapies that not only target specific tumor antigens but also reverse the immunosuppressive BM microenvironment have shown promise. The anti-CD38 monoclonal antibodies (MoAbs) daratumumab and isatuximab, which target MM cells and block immunosuppressive regulatory cells (i.e., Treg, Breg, myeloid-derived suppressor cells), have a significant treatment response in RRMM patients (16, 17). In addition, immunotherapies based on chimeric antigen receptor (CAR)-T cells, which are T cells engineered with a particular T cell receptor, have an impressive overall response rate (ORR) in several clinical trials. The high rates of response and minimal residual disease (MRD) negativity led to the approval of idecabtagene vicleucel (Abecma) in 2021 and ciltacabtagene autoleucel (Carvykti) in 2022 by the US Food and Drug Administration (FDA) for the treatment of heavily pretreated RRMM patients.

The clinical success of anti-BCMA CAR-T cell therapy prompted further development of different T-cell-directing immunotherapies, such as bispecific antibodies (BiAbs) or bispecific T-cell engagers (BiTEs). BiAb and BiTE commonly target both CD3 on T cells and tumor-associated antigens on the surface of MM cells, resulting in MM cell killing mainly *via* the release of perforins and granzymes from the T cells (27). In addition, these bispecific molecules also mediate T-cell activation and proliferation. The immunomodulatory effect of these agents is independent of antigen presentation on the major histocompatibility complex (MHC) class I, can occur in the absence of co-stimulation, and bypass the normal dependence on antigen-presenting cells or cytokines (28, 29), making them suitable for use in the dysfunctional immune systems of MM patients. In fact, some of these therapies -continue to show encouraging results in early-phase trials in RRMM patients (30–32).

## Mechanisms of action of BiTEs and BiAbs

**Figure 1:**
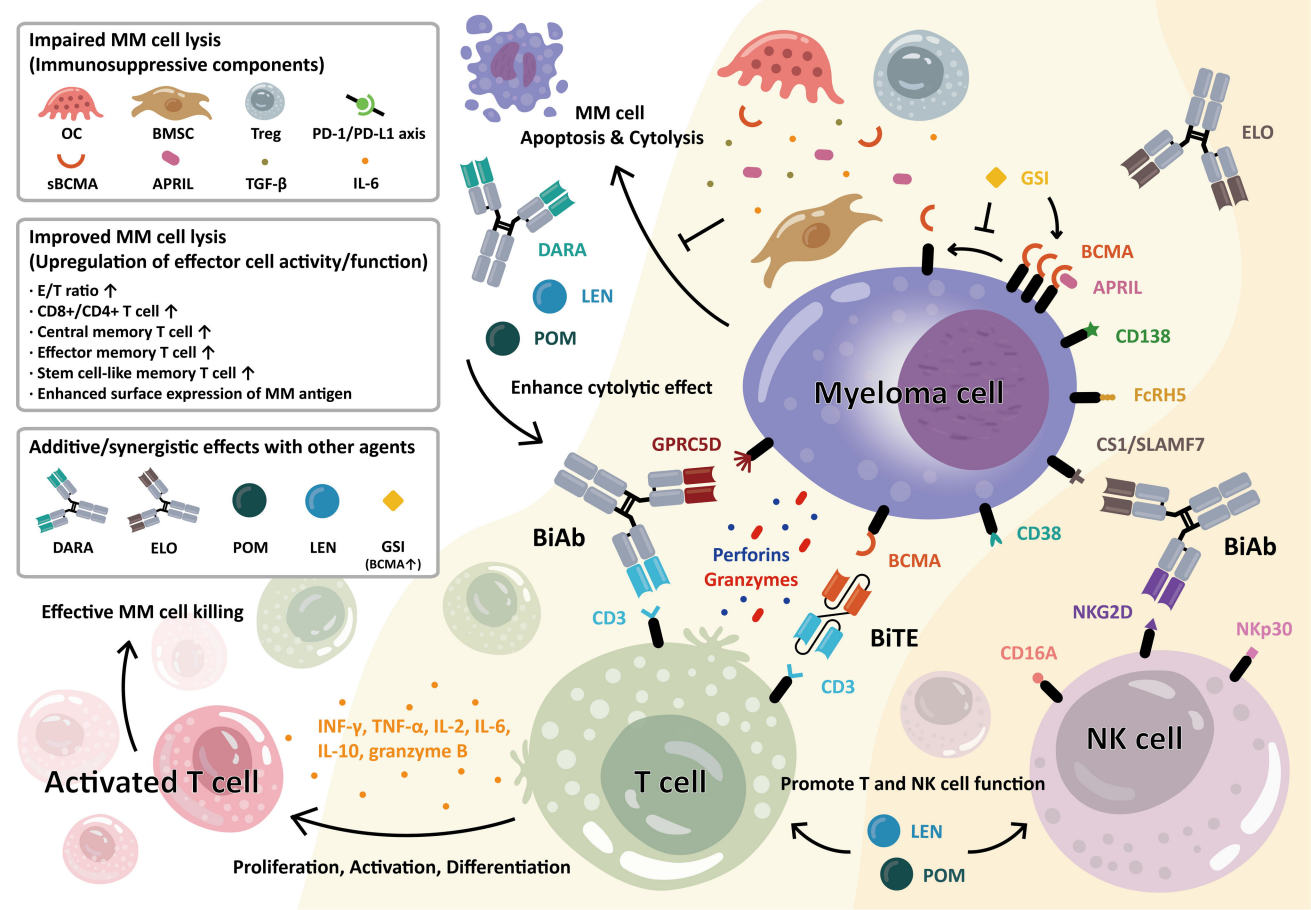
Anti-myeloma activity of bispecific antibody (BiAb) and bispecific T-cell engager (BiTE) molecules in the bone marrow MM microenvironment. T-cell–redirecting BiAb and BiTE simultaneously bind to the myeloma-specific antigens on MM cells and CD3 on T cells. MM antigens include BCMA, CD38, CS1/SLAMF7, GPRC5D, and FcRH5, as indicated. Upon engagement, the immune synapse is formed, followed by the production and secretion of cytolytic molecules, i.e., perforin and granzymes, from T cells, resulting in MM cell lysis. This further induces T-cell activation, proliferation, and differentiation into various memory subsets. The BiAb/BiTE-mediated T-cell activation leads to increased levels of granzyme B, IFN-γ, IL2, IL6, IL8, IL10, and TNF-α. The BiAb/BiTE-mediated MM cell killing is negatively affected by cellular and molecular factors including bone marrow stromal cells (BMSC), osteoclast (OC), regulatory T cells (Treg), a proliferation-inducing ligand (APRIL), transforming growth factor-β (TGF-β), interleukin-6 (IL-6), soluble BCMA (sBCMA), and upregulation in PD-L1/PD1 axis. Conversely, upregulation in effector/target (E/T) ratio, CD8+ T cell, and differentiated T cells with central and stem-like memory subsets are associated with improved BiAb/BiTE-mediated MM cell lysis. Furthermore, the potency and durability of their ability to kill MM cells could be enhanced when combined with current standard-of-care therapies including daratumumab (DARA), elotuzumab (ELO), lenalidomide (LEN), or pomalidomide (POM). Moreover, soluble BCMA (sBCMA), constantly shed by gamma-secretase (GS), could antagonize optimal MM cell eradication by BCMA-targeting agents. The GS inhibitor (GSI) rapidly blocks the release of sBCMA and augments BCMA protein retention on the MM cell membrane, thereby MM cell targeting and killing are significantly improved. In a similar manner, the BiAbs or natural killer cell engagers (NKCEs) also target natural killer (NK) cell-related receptor antigens (i.e., CD16A, NKG2D, NKp30) to activate NK cells and augment their anti-MM activities. For example, the anti-CS1 Ab elotuzumab (ELO) enhances NK cell function *via* CS1 and NKG2D to kill MM cells. (Some elements of **Figure 1** are created with BioRender.com).

The structure and function of BiTEs and BiAbs are somewhat similar, in that they both have two binding sites that either bind two different antigens or two epitopes of the same antigen. BiAbs are engineered artificial antibodies, whereas BiTEs are recombinant proteins composed of two linked scFvs (single-chain variable fragment). Either way, they can simultaneously bind a tumor cell and an immune effector cell, creating an immune synapse between the tumor cell and T cell, which encourages T cell activation, tumor cell lysis, and T cell proliferation (33, 34). The killing effect of BiTE involves polyclonal T cell responses, which are independent of recognition and costimulation *via* the MHC and T cell receptor (TCR) (35). In fact, when T cells are incubated with tumor cells in the presence of BiTEs, both CD8+ and CD4+ T cells become activated, followed by significant tumor cell lysis, with more pronounced MM cell killing by CD8+ over CD4+ T cells (36). Moreover, these bispecific molecules modulate general T cell function. For example, BiTEs and BiAbs targeting CD3 and BCMA induce T-cell-mediated tumor cell killing accompanied by significantly increased expression of T-cell-activation-related parameters (27, 34, 37), and the differentiation of naïve T cells to T cells with memory phenotypes (central memory and effector memory T cells) (34), which could contribute to improved MRD negativity. The anti-MM activity of BiTEs and BiAbs in the MM BM microenvironment is shown in **Figure 1**.

## BiTEs and BiAbs in MM treatment by therapeutic target

### B-cell maturation antigen (BCMA, CD269, TNFRS17)

BCMA, a member of the tumor necrosis factor superfamily (TNFRSF17), is highly expressed only on the surface of plasma cells and somewhat on plasmacytoid dendritic cells, making it an ideal target for immunotherapy in MM (38). BCMA binds to APRIL, which is predominantly secreted by monocytes and myeloid-linage cells (macrophages and osteoclasts), and together, they regulate plasma cell proliferation and long-term survival in healthy contexts and MM cell growth, MM cell survival, drug resistance and an immunosuppressive MM BM microenvironment (25). Gamma (γ)-secretase cleaves membrane BCMA (mBCMA) into soluble BCMA (sBCMA), which is released into circulation (39). MM patients have significantly higher serum sBCMA levels than healthy individuals, and these levels are associated with increased MM burden and poorer overall and progression-free survival, suggesting that sBCMA levels represent a biomarker for MM (40). Anti-BCMA therapy has shown impressive results in several early-phase clinical trials, and subsequent trials led to the FDA approval of the first CAR-T cell therapy in treating RRMM. Now, the next generation of immunotherapy, BiTEs and BiAbs, are targeting this promising protein.

#### Pacanalotamab (AMG 420, BI 836909)

Pacanalotamab (AMG 420, BI 836909) is the first BiTE developed for myeloma treatment, and it binds both BCMA (on MM cells) and CD3 (on cytotoxic T cells). Pacanalotamab effectively lysed BCMA-expressing myeloma cells in *in vitro* and *in vivo* preclinical models (33). In *ex vivo* co-cultures of MM-PBMC or T cells, it induced significant T cell proliferation, yet sBCMA at higher concentrations reduced its killing of MM cells in *in vitro* cytotoxicity assays. In the subsequent phase 1 study (NCT02514239), pacanalotamab was administered to 42 patients with RRMM (≥ 2 prior lines) at various doses (0.2-800 μg/d). Cytokine release syndrome (CRS) was observed in 16 patients, mostly at grade 1 or 2. The ORR was 70%, including 50% MRD-negative complete responses (CR) at the dose of 400 μg/d, the maximum tolerable dose (MTD) for this study (41). These results are comparable to CAR-T cell therapy and the low rates of neurotoxicity are promising, yet it had a somewhat high incidence of serious infections (33%), which would need further development to combat (41).

#### Pavurutamab (AMG 701)

Pavurutamab (AMG 701) is similar to pacanalotamab, but it has an Fc moiety on the anti-CD3 domain that extends its serum half-life to 112 hours, making weekly dosing a possible approach, as compared to pacanalotamab, which needs a continuous IV infusion (42). In addition, the 3-dimensional model suggests that pavurutamab forms an immunological synapse of ~100 Å between the tumor cell and T cell (43). In preclinical studies, pavurutamab demonstrated potent anti-MM activity in co-culture models, including with autologous patient MM cells, and it induced the robust activation and proliferation of CD4+ and CD8+ T cells as well as the differentiation of memory T cells. The anti-MM effect could be further enhanced by adding IMiDs (lenalidomide or pomalidomide) or immune checkpoint inhibitors including an anti-programmed cell death protein-1 (PD-1) antibody (34, 43).

In the first-in-human phase 1 study (NCT03287908), 75 heavily pretreated RRMM patients (≥ 3 prior lines) received pavurutamab monotherapy with differing doses (0.14–12mg). A SAE was noted in 29 patients including infection (n = 13) and CRS (n = 7), with a milder form of CRS in 53% of patients (grade 1 or 2). The response rate was 36% at doses between 3–12 mg (16/45). At the dose of 9 mg, the response rate was 83% (earlier dose escalation, 5/6), with one response at 0.8 mg for a patient with a low baseline of sBCMA (MRD-negative CR). The minimal residual disease (MRD) analysis was done in 4 CR patients (3 stringent CR [sCR], 1 CR), and the results were all negative (32). Overall, it demonstrated a manageable safety profile, encouraging activity, and favorable pharmacokinetic profile. However, in 2022, the clinical development of pavurutamab was discontinued due to a strategic decision of the developer (**Supplement Table 1**).

#### Teclistamab (JNJ-64007957)

Teclistamab (JNJ-64007957) is a humanized BCMA/CD3 bispecific IgG4 antibody (27). In preclinical studies, teclistamab showed potent anti-MM activity against MM cells lines and myeloma cells from MM patients. Soluble BCMA decreased the ability of teclistamab, which could be overcome with higher teclistamab concentrations. In addition, pretreatment or combination with anti-CD38 MoAb, such as daratumumab, or a γ-secretase inhibitor further enhanced MM cell lysis (37).

In a phase 1/2 trial (Majes TEC-1, NCT03145181, and NCT04557098), a total of 165 RRMM patients (at least 3 prior treatment lines) received teclistamab intravenously (range 0.3−19.2 μg/kg [once every 2 weeks] or 19.2−720 μg/kg [once weekly]) or subcutaneously (range 80−3000 μg/kg [once weekly]). The ORR was 63% (104/165), including 97 patients who achieved a very good partial response (VGPR) or better and 65 patients achieving CR or better. The median progression-free survival (PFS) and overall survival (OS) were 11.3 and 18.3 months, respectively. CRS was observed in 119 patients (72.1%), with most being grade 1 or 2 in severity. Neurotoxic events, including immune effector cell–associated neurotoxicity syndrome, were found in 24 patients (14.5%), and no patients discontinued teclistamab treatment because of neurotoxicity (44). Overall, teclistamab induced durable responses that deepened over time, and it was recently approved by the European Medicines Agency (EMA) for the treatment of adult patients with RRMM. Teclistamab (TECVAYLI) is thus the first T-cell redirecting BiAb available for adults with RRMM.

#### Elranatamab (PF-06863135)

Elranatamab (PF-06863135) is a humanized anti-BCMA/CD3 bispecific IgG2a antibody (45). In preclinical studies, elranatamab was effective as a single agent in *ex vivo* 3D primary MM cultures (EC_50_ = 0.2 nM) and could be augmented with anti-PD-1 or IMiDs including lenalidomide. Furthermore, pretreatment with γ-secretase inhibitor (GSI) inhibited the formation of soluble BCMA, which promoted MM cell lysis.

In a phase 1 clinical trial (MagnetisMM-1 trial, NCT03269136), 58 patients with RRMM received weekly subcutaneous elranatamab administration, either alone (n = 50), with lenalidomide (n = 4) or with pomalidomide (n = 4) (46). CRS was observed in 48 patients (83%, none higher than grade 2). In terms of efficacy, there were 14 patients with confirmed responses, including 5 sCRs, 1 CR, 7 VGRPs, and 1 PR. The ORR in patients who received the recommended Phase 2 dose was 83% (5/6). The updated data was reported in 2022 (47). Among 55 patients who received elranatamab with a dose of ≥ 215 μg/kg, the ORR was 64%, with 31% of patients achieving CR or better. CRS was observed in 67% of patients who received the recommended dose (1000 µg/kg or 76 mg), with no grade 3 or higher CRS. Overall, due to its manageable safety profile and durable responses, elranatamab was granted an Orphan Drug Designation by the FDA and EMA for the treatment of MM.

#### Linvoseltamab (REGN5458)

Linvoseltamab (REGN5458) is a fully humanized BCMA/CD3 BiAb, which is generated by Regeneron’s proprietary ‘human antibody mouse’ technology (VelocImmune) and ‘full-length BiAb’ platform (VelociBiTM) (48, 49). In pre-clinical studies, linvoseltamab potently induced T-cell mediated killing of MM cell lines and primary human plasma cells, as well as T cell activation and cytokine production. The binding of linvoseltamab to MM cells led to increased surface BCMA accumulation on MM cell lines, and in animal model experiments, linvoseltamab showed similar anti-MM activity as anti-BCMA CAR-T cells but with different kinetics.

In the phase 1/2 first-in-human clinical trial (LINKER-MM1) (NCT03761108) reported recently, 73 RRMM patients (at least 3 lines of prior therapy) in the dose-escalating cohort received linvoseltamab treatment (full dose: 3–800 mg) (50). Treatment-related AEs were reported in 73 patients (100%), including 55 patients (75.3%) who experienced grade 3 or higher AE. Common AEs included fatigue (45.2%) and CRS (38.4%, no ≥ grade 3). Nausea was noted in 24 patients (32.9%). Treatment responses were noted at all dose levels. Among the patients at the 200–800 mg dose levels, the response rate was 75.0% (18/24). Overall, it showed a manageable safety and tolerability profile (lower severe CRS rate than other anti-BCMA BiAbs), with early, deep, and durable responses.

#### TNB-383B (ABBV-383)

TNB-383B (ABBV-383) is a next-generation fully human bispecific monoclonal IgG4 antibody targeting BCMA and CD3 (51). In preclinical studies, TNB-383B showed potent anti-MM activity and T-cell activation in *in vitro* and *ex vivo* experiments. It significantly reduced tumor burden and increased host survival in animal models. TNB-383B had a half time of ~13–16 days in cynomolgus monkey, consistent with an IgG4 Ab. TNB-383B also induced T-cell-mediated lysis of primary MM cells from relapsed patients, with a higher percentage of lysis and cytotoxic T lymphocyte degranulation in higher BCMA-expressing MM cells (52).

In the phase 1 trial (NCT03933735), 124 patients with RRMM (≥ 3 prior lines), including 73 in the dose escalation cohort (dose: 0.025–120 mg) and 51 in the dose expansion cohort (60 mg), received TNB-383B treatment (53). The most common AEs was CRS (n = 71, 57%). In the dose-escalation cohorts (≥ 40 mg Q3W, n = 79), the response rate was 68% (54/79), with a VGPR or better rate of 54% (43/79), and a CR rate (CR + sCR) of 36% (29/79). In patients with triple-class refractory disease treated with a dose ≥ 40 mg (n= 64) or 60mg (n= 41), the ORR were 63% and 54%, respectively. Overall, the treatment was well tolerated, and though the follow-up period was short, the ORR rates are promising.

#### Alnuctamab (CC-93269, EM 801)

Alnuctamab (CC-93269, EM 801) is a BiAb with 2 asymmetric arms carrying humanized IgG1 T-cell engagers that bind bivalently to BCMA and monovalently to CD3ϵ in a 2 + 1 format (38). In preclinical studies, alnuctamab induced robust MM cell death at nanomolar concentrations, activation of CD4+/CD8+ T cells, and production of IFN-γ, granzyme B, and perforin. In an autologous model, alnuctamab also induced lysis of BCMA+ cells, including those from high-risk or RRMM patients.

In the first-in-human phase 1 trial (NCT03486067), 19 patients with RRMM received weekly alnuctamab administration (doses from 0.15 mg to 10 mg) (54). Most of the patients (n=15, 78.9%) experienced treatment-related grade 3 to 4 AEs. CRS was observed in 89.5% of patients, resulting in one death in this trial. In terms of clinical efficacy, no clinical response was observed in patients who received <3mg of alnuctamab, but at doses of ≥6 mg, the ORR was 83.3%. Overall, the authors claim alnuctamab has a manageable safety profile and promising efficacy, though the safety profile appears at first glance to be worse than the other candidates.

### G-protein coupled receptor family C group 5 member D (GPRC5D)

GPRC5D is a recently identified MM antigen that is highly expressed on malignant MM plasma cells in the BM and lowly expressed on hair follicles, but not on other healthy cells (55). Notably, the expression of GPRC5D on CD138+ MM cells is independent of BCMA, supporting that GPRC5D is a novel MM antigen. Specifically, in a preclinical study, anti-GPRC5D CAR-T cells demonstrated potent and selective MM cell killing (55), validating GPRC5D as a promising target for BiAbs and BiTEs.

#### Talquetamab (JNJ-64407564)

Talquetamab (JNJ-64407564) is the first bispecific IgG4 antibody targeting GPRC5D on MM cells and CD3 on T cells (56, 57). In preclinical studies, talquetamab induced activation and degranulation of T cells, as well as lysis of MM cells collected from newly diagnosed or relapsed MM patients. It also induced T cell activation–related cytokines, including IFN-γ, TNF-α, IL-2, and IL-10, but its anti-MM activity was adversely affected by Treg, BMSC, or T cells expressing a high level of PD1 or HLA-DR. Combining it with daratumumab or pomalidomide enhanced its MM cell lysis activities.

In the phase 1 MonumenTAL-1 trial (NCT03399799), patients with RRMM received subcutaneous talquetamab treatment (405 μg/kg QW [n = 30]/800 μg/kg Q2W [n = 44]) (58). The most common treatment-related AEs were cytopenias (67%/36%) and CRS (77%/80%, no grade 3). Skin-related and nail disorders were found in 83%/75% of patients (most common: skin exfoliation: 37%/39%, all grades 1 and 2). Regarding clinical efficacy, the ORRs were 70% (21/30)/64% (28/44). Because talquetamab showed substantial clinical efficacy, the FDA granted it a Breakthrough Therapy Designation (BTD) in July 2022 for the treatment of adult patients with RRMM who have previously received at least 4 prior lines of therapy, including a proteasome inhibitor, an immunomodulatory agent, and an anti-CD38 antibody.

#### RG2634 (RO7425781)

RG2634 (RO7425781) is a BiAb with a novel 2:1 format for anti-GPRC5D:anti-CD3 (59). RG2634 is also characterized by a silent Fc region that reduces toxicity and increases its half-life. In the phase 1 trial (NCT04557150), 41 patients with RRMM received intravenous or subcutaneous infusions of RG2634 in a step-up dosing regimen. CRS occurred in 85.4% of patients, generally confined to the first cycle. Three patients (7.3%) experienced CNS toxicity related to RG6234. Skin-related AEs were observed in 66% of patients (Grade 3: 7.3%). After the first cycle, 93% of evaluable patients (14/15) had <1% of MM cells in BM based on flow cytometry. The ORR in 34 efficacy-evaluable patients across all doses was 68%, with 50% achieving a VGPR or better response. These early promising data could further clinical development of this novel agent in treating patients with MM, alone or in combination.

#### GPRC5D TRAB

GPRC5D TRAB is an anti-GPRC5DxCD3 bispecific T-cell–redirecting IgG-based antibody (TRAB) (60). In preclinical studies, GPRC5D was confirmed as an MM-specific antigen. Then four Mabs targeting GPRC5D were generated (GPA0018, GPA0021, GPA0032, and GPA0039), with GPA0018 and GPA0039 demonstrating potent T-cell activation and cytotoxic effects on MM cells in *in vitro* and *in vivo* models. In a mouse model, the gene expression profile showed the genes related to immune activation were upregulated. These data further support clinical development of GPRC5D TRAB.

### Fc Receptor Homolog 5 (FcRH5, FcRL5, CD307)

FcRH5 is a type I membrane protein that is selectively expressed on B cells and plasma cells. The expression levels of FcRL5 are variable but are generally elevated on BM plasma cells derived from MM and MGUS patients compared with plasma cells from normal individuals (58). Immunotherapy targeting FcRH5 was once evaluated in a phase 1 trial (NCT01432353), with the antibody-drug conjugate (ADC) DFRF4539A showing limited anti-myeloma activity in RRMM patients. Despite low ORR, this study revealed FcRH5 was widely expressed on MM cells and can be occupied by antibodies after DFRF4539A treatment (61). The investigations of BiAb targeting FcRH5 are ongoing.

#### Cevostamab (BFCR4350A)

Cevostamab (BFCR4350A) is an IgG-based T-cell-engager BiAb, which is able to target the most membrane-proximal domain of FcRH5 on MM cells and CD3 on T cells (62). In preclinical studies, it effectively eradicated human MM cell lines and primary myeloma cells at picomolar concentrations. Robust T cell activation and proliferation were also noted. In animal model experiments, complete depletion of B cells and bone marrow plasma cells was observed in cynomolgus monkeys. Importantly, blockade of PD-1/PD-L1 by an anti-PD-L1 agent enhanced the activity of BFCR4350A.

The results of 51 patients with RRMM in a dose-escalating cohort were reported in 2020 in the phase 1 trial (GO39775 study, NCT03275103) (63). Common treatment-related AEs included CRS (74.5%, mainly grade 1/2), neutropenia (11.8%), and lymphopenia (11.8%). In terms of efficacy, 46 patients were evaluated. Treatment responses were observed at the 3.6/20mg dose level and above in 15 patients (15/29, 51.7%), including 3 sCRs, 3 CRs, 4 VGPRs, and 5 PRs. At the 3.6/20 mg dose level and above, responses in patients with high-risk cytogenetics, triple refractory disease, and prior anti-CD38 treatment were 52.9% (9/17), 50% (10/20), and 50% (11/22), respectively.

The updated data of the GO39775 study was reported in 2021 (64). The most effective doses for single step-up (SS) and double step-up (DS) administration were 3.6 mg and 0.3/3.6 mg, respectively. The incidence of CRS was similar. The incidence rate of CRS was lower in the patients who received the 0.3/3.6 mg DS regimen (77.3% vs 88.2%). In the dose-escalation cohort, clinical responses were observed at the 20–198 mg target dose levels. The clinical efficacy of two dose-expansion cohorts was investigated (90 mg, n = 44; 160 mg, n = 60), with higher ORR in the 160 mg cohort vs the 90 mg cohort (54.5% vs 36.6%). The estimated median duration of response was 15.6 months. Overall, cevostamab showed clinically meaningful activity, with a dose-dependent increase in ORR (without an increase in CRS rate) and responses that appear durable.

### CD38

CD38 is a type II transmembrane glycoprotein first identified as a marker of cell activation and proliferation in 1990. In the BM of MM, CD38 is highly expressed on the surface of myeloma cells and is closely associated with the immunocompromised tumor microenvironment (65, 66). MM cells can utilize aerobic glycolysis to promote an acidic BM, which together with CD38, promotes the generation of AMP and adenosine, a molecule with immunosuppressive activity. Recently, a study revealed CD38 was downregulated by IL-6, which is secreted by BMSCs *via* activation of IL-6-induced JAK1/2-STAT1/3 signaling pathways, and this can be reversed by JAK1/2 inhibitor (67, 68). The success of anti-CD38 MoAb daratumumab and isatuximab led to more studies exploring bispecific antibodies targeting CD38.

#### ISB 1342 (GBR 1342)

ISB 1342 (GBR 1342) is the first CD38/CD3 BiAb engineered (using the Glenmark Bispecific Engagement by Antibodies based on the T cell receptor [BEAT] platform) to direct T cells to CD38-expressing myeloma cells, leading to MM cell lysis (69). Importantly, the fragment antigen-binding arm of ISB 1342 specifically recognizes CD38 and does not compete with daratumumab. In preclinical studies, ISB 1342 effectively induced robust CD38+ MM cell lysis (EC_50_: 12 to 90 pM) and overcame the resistance to anti-CD38 MoAbs by binding to a different CD38 epitope. Furthermore, ISB 1342 showed better *in vivo* anti-MM activity than daratumumab in an animal study. The phase 1 trial of ISB 1342 in RRMM patients is ongoing (NCT03309111).

#### AMG 424

AMG 424 is a humanized T cell-recruiting BiAb targeting CD3 and CD38 that contains an XmAb Fc domain and cross-reacts with nonhuman primate CD3 and CD38 (70), allowing studies in monkeys. In preclinical studies, AMG 424 induced CD38+ MM cell lysis, as well as T-cell activation and proliferation. AMG 424 had potent antitumor activity in BM–invasive cancer mouse models and depleted peripheral B cells in a cynomolgus monkey model. Despite its therapeutic potential, the clinical trial of AMG 424 (NCT03445663) was terminated by the sponsor.

#### Other anti-CD38 bispecific formats

The anti-CD38/CD3 BiAb developed by Sorrento Therapeutics induced robust T cell-dependent lysis of CD38+ cancer cells *in vitro* (71) and was a more potent tumor cell killer than daratumumab. BI38-3, a BiTE reported in 2021, induced T-cell-mediated cytotoxicity on CD38+ MM cells (72), specifically against cells expressing high levels of CD38, whereas cells with weaker CD38 expression were insensitive, including hematopoietic progenitor cells, B cells, T cells and NK cells.

### CD138 (Syndecan-1)

CD138, belonging to the syndecan family of type I transmembrane proteoglycan, is highly and specifically expressed on the surface of MM cells. Increased expression of CD138 supports the proliferation and survival of MM cells, as well as angiogenesis and IL-6 receptor sensitivity in MM cells (73). CD138 is cleaved by metalloproteinases and heparinase to form soluble CD138 (sCD138), whose serum levels are correlated with disease burden and prognosis of MM (74). The first anti-CD138 immunotherapeutic agent was an antibody-drug conjugate, BT062/Indatuximab ravtansine (75, 76), which had encouraging results alone or combined with other MM agents in clinical trials. Recently, a new anti-CD138 MoAb VIS832 showed enhanced membrane CD138-binding affinity, resulting in significant anti-MM activity in *in vitro* and *in vivo* preclinical models (77). When combined with bortezomib, complete MM eradication was seen in a xenograft murine model, suggesting the potential combination use of VIS832 with bortezomib in MM.

Regarding the development of anti-CD138/CD3 agents, some preclinical studies have shown encouraging results. First, STL001 (BiTE-hIgFc), a BiTE with two scFv arms and an IgG1 Fc region for binding NK cells, induced T-cell activation and MM cell lysis (78). Another study showed that BiAbs h-STL002 and m-STL002 also exhibit potent cytotoxic effects against MM cells *via* T cell activation (79).

## Beyond bispecific antibodies: Trispecific antibodies and natural killer cell engagers

The above BiAbs and BiTEs target T cells. However, bispecific formats targeting natural killer (NK) cells are also under clinical development. Indeed, a novel form of TriAbs characterized by a combination of a single-chain Fv against CD16 and two tumor-associated antigens has shown promising anti-tumor activity in preclinical studies (80). These molecules activate NK cells *via* CD16 to augment NK cell cytotoxic activity and cytokine production to kill tumor cells by targeting specific antigens. Other novel bispecific/trispecific formats are under rigorous investigation. For example, the BCMA/CD200/CD16A specific antibody is a tetravalent ‘aTriFlex’ TriAb characterized by a fusion protein bivalently engaged to CD16A on NK cells and two low-affinity binding single-chain antibodies targeting BCMA and CD200 on MM cells (81). The dual-targeting design may increase the specificity to MM cells and improve safety. In preclinical studies, the BCMA/CD200/CD16A-specific antibody demonstrated potent and specific anti-MM activity.

Another format, AFM26, targeting BCMA and CD16A, is the first bispecific NK-cell engager (82). A novel BiAb was also developed to target another MM-specific antigen, CS1 (SLAMF7), and NKG2D on cytolytic immune cells (including NK cells, CD8+ T cells, γδ T cells, and NK-T cells), leading to effective MM lysis (83). In addition, 2A9-MICA is a novel BiAb targeting BCMA and MICA, which effectively activated induced NK cells to kill BCMA+ human myeloma cells in a preclinical study (84). CTX-8573 is a BiAb targeting BCMA and NKp30, characterized by an IgG1-like afucosylated Fc region to additionally bind to CD16A on NK cells and γδ-T cells (85), leading to potent cytotoxicity of BCMA+ cells in preclinical studies. CTX-8573 also demonstrated anti-tumor efficacy in humanized mouse models and displayed standard biphasic pharmacokinetics, with a 16-day β-phase half-life in cynomolgus monkey.

A novel idea for TriAb design is to stimulate T cells *via* two T cell antigens (not only CD3) to enhance activation (86). CD28, a well-known second signal on T cells, has been utilized as the costimulatory domain in CAR-T cell therapy. A preclinical study evaluating an anti-CD3, CD28, and CD38 TriAb revealed that targeting CD3 and CD28 enhanced T cell activation and prolonged survival (87). In an animal model, this TriAb further suppressed MM growth, stimulated memory/effector T cell proliferation and reduced the number of Tregs.

## Using BiAbs and BiTEs in combination therapies

BiAb and BiTE treatment approaches have produced remarkable anti-MM effects in heavily pretreated patients with RRMM. To further optimize BiAb/BiTE treatment, several novel strategies to augment their potency are under investigation. For example, a high MM tumor burden adversely affects the potency of BiAb, which is related to T cell exhaustion, but it can be overcome by combining it with another cytotoxic chemotherapy (88). Moreover, the combination of BiAb/BiTE with another immunotherapeutic agent, including IMiDs or anti-PD1 agents, may have synergistic anti-MM effects with prolonged responses (34, 43, 56). In addition, combining it with an anti-CD38 MoAb, such as daratumumab and isatuximab, may improve response by overcoming the immunosuppressive BM microenvironment (16, 17). For example, subcutaneous teclistamab combined with daratumumab in RRMM patients (TRIMM-2 study, NCT04108195) caused a rapid treatment response and a high response rate (78%, 29/37), including 73% with VGPR or better. The most common AE included CRS (54.5%, all grades 1 and 2), neutropenia (36.4%), and thrombocytopenia (36.4%), which were all manageable (89). A phase 3 trial evaluating teclistamab/subcutaneous daratumumab versus daratumumab/pomalidomide/dexamethasone or daratumumab/bortezomib/dexamethasone in RRMM is ongoing (MajesTEC-3 trial, NCT05083169).

With respect to other anti-BCMA/CD3 bispecific molecules, several trials investigating combination strategies are also ongoing in patients with RRMM. The combination of pavurutamab (AMG 701) with pomalidomide and dexamethasone is being evaluated in patients with RRMM post ≥ 3 lines of prior treatments (NCT03287908). Elranatamab (PF-06863135) monotherapy or combined with daratumumab versus daratumumab/pomalidomide/dexamethasone is also being evaluated in patients with RRMM (phase 3 MagnetisMM-5 study, NCT05020236). Furthermore, the combination of REGN5458 with other anti-MM agents is also under investigation (NCT05137054).

## Comparing BiAbs and BiTEs to existing immunotherapies

Following the success of BCMA-targeting antibody-drug conjugates (ADC) and CAR-T cell therapies, BiAb/BiTEs represent additional effective mono-immunotherapies for MM, with impressive clinical activity and acceptable safety in various early-phase clinical trials (**Table 1** and **Supplemental Table S1**). Compared with ADC and CAR-T cell therapy, bispecific molecules have unique characteristics that make them stand out as another feasible therapeutic option. For example, CAR-T cells are engineered from living cells, which may take a month (median: 34, range: 33-68) according to the KarMMa study (NCT03361748) with a manufacturing failure rate of ~10% (90). Some patients with a rapid progression of MM may need bridging therapy or to withdraw in favor of another available treatment (91, 92). On the other hand, BiAb/BiTE molecules are off-the-shelf agents, providing superior availability and manufacturing reliability.

**Table 1:** Summary of clinical trials of BiTE/BiAb therapy.

Agents (target)	Trial and participant data	Protocol	Efficacy	Safety
Teclistamab (JNJ-64007957) BCMA x CD3	1. Phase 1/2 (NCT03145181 and NCT04557098). 2. RRMM, n= 165. 3. Median age: 64 years (range: 33-84). 4. Prior lines of Tx: 5 (2-14) 5. EMD: 17%. 6. Prior HSCT: 81.8% 7. Refractory to anti-CD38 MoAb: 89.7%. 8. Triple/Penta refractory: 77.6%/30.3%.	1. Once-weekly SC teclistamab at a dose of 1.5 mg/kg (step-up doses of 0.06 and 0.3 mg/kg).	1. ORR: 63% (104/165), including 97 ≥ VGPR, 65≥ CR. 2. Median time to the first/best response: 1.2/3.8 months. 3. MRD- (10^-5^): 26.7% (44/165). 4. mPFS/mOS: 11.3/18.3 months.	1. AEs (any/Gr 3 or 4): 100%/94.5%. 2. Neutropenia (70.9%), anemia (52.1%), thrombocytopenia (40%). 3. Infection: 76.4%, including 44.8% of Gr 3/4. 4. Hypogammaglobulinemia: 74.5%. 5. CRS: 72.1% (most Gr 1 or 2). 6. Neurotoxicity: 14.5% (most Gr 1 or 2), including headache (8.5%).
Elranatamab (PF-06863135) BCMA x CD3	1. Phase 1 (MagnetisMM-1, NCT03269136) 2. RRMM, n=58 3. Median age: 64 years (range: 32-86). 4. Prior lines of Tx: 6 5. Triple refractory: 98% 6. Prior HSCT: 45%. 7. Prior BCMA-targeted Tx: 22%. 8. Black/African American or Asian: 26%.	Dosing: Part 1: 80, 130, 215, 360, 600, and 1000μg/kg weekly Parts 1.1 and 2A: single 600μg/kg or equivalent fixed dose of 44mg first, followed 1 week later by 1000μg/kg or equivalent fixed dose of 76mg weekly (Q1W) or every 2 weeks (Q2W) thereafter. LEN or POM combination therapy: single priming dose (32mg), followed 1 week later by the 44mg Q1W thereafter plus LEN (25mg) or POM (4mg) on Days 1 to 21 of a 28-day cycle	14 patients with confirmed responses. 1. ORR Part 1 (215-1000μg/kg): 70% (14/20), including 6 CR/sCR. At RP2D dose: 83% (5/6) Prior BCMA-targeted Tx: 75% (3/4). 2. Median time to response: 22 days.	TEAEs: 1. CRS: 48 (83%), none higher than Gr 2. 2. Lymphopenia (n=37, 64%; Gr 3/Gr 4:12%/52%), neutropenia (n=37, 64%; Gr 3/Gr 4:31%/29%), anemia (n= 32, 55%; Gr 3/Gr 4: 38%/0%), injection site reaction (53%), and thrombocytopenia (52%). 3. 2 DLT: 1 Gr 4 thrombocytopenia (part 1.1), 1 Gr4 neutropenia (POM).
Linvoseltamab (REGN 5458) BCMA x CD3	1. Phase 1/2 (NCT03761108). 2. RRMM, n=73. 3. Median age: 64 years (range: 42-81). 4. 20.5% patients≥ 75 years). 5. Prior lines of Tx: 5 (2-17). 6. Penta refractory: 38.4%. 7. Prior auto-HSCT: 64.4%.	1. Weekly doses of REGN5458, followed by a maintenance phase administered every 2 weeks (doses ranging from 3-800 mg).	1. ORR: At 200-800 dose levels: 75.0% (18/24) At all dose levels, 86.5% (32/37) of all responders achieved VGPR and 43.2% (n=16) of responders had a CR or SCR. 2. Estimated DOR ≥8 months: 90.2%	1. AEs: 73 (100%), including 31 Gr 3 (42.5%) and 24 Gr 4 (32.9%). 2. Fatigue (n= 33, 45.2%), CRS (n= 28, 38.4%) 3. CRS: Gr 1 in 25 pts and Gr 2 in 3 pts. 4. No Gr ≥3 neurotoxicity events 5. Nausea: 24 (32.9%)
TNB-383B (ABBV-383) BCMA x CD3	1. Phase1 (NCT03933735). 2. RRMM, n=124. (ESC, n=73; ESP (60mg), n=51) 3. Median age: 68 years (range: 35-92). 3. Prior lines of Tx: 5 (3-15). 4. Triple/penta-refractory: 82%/35 (≥ 40mg: 81%/41). 5. Prior HSCT: 81% (≥ 40mg ESC+ EXP: 83%).	1. IV infusion over 1–2 hours every 3 weeks (Q3W). 2. Dose range: 0.025–120 mg 3. RP2D: 60mg	Evaluable, n=122 1. ORR: ≥ 40mg ESC + EXP (n=79): 68%, 43 ≥VGPR (54%), 13CR, 16sCR. 60mg EXP: 59% (29/49), 19 ≥VGPR (39%), 7CR, 4sCR. 2. PFS All: 10.4 months ≥ 40mg ESC + EXP/60mg EXP: NR/NR	Evaluable, n=124 1. Three DLTs (Gr 4 thrombocytopenia, 60mg; Gr 3 CRS, 90 mg and 120 mg) 3. TEAEs: CRS (n= 71, 57%), neutropenia (n= 46, 37%), and fatigue (n= 37, 30%). 3. Infection (≥ Gr3): Pneumonia, COVID-19, sepsis (all n=19, 6%) 4. Seven deaths from TEAEs.
Alnuctamab (CC-93269) BCMA x CD3	1. Phase1 (NCT03486067). 2. RRMM, n=19 (received Tx). 3. Median age: 64 years (range: 51-78). 4. Prior lines of Tx: 6 (3-12). 5. Prior auto-HSCT: 73.7%. 6. Prior allo-HSCT: 10.5%. 7. Prior lenalidomide/bortezomib: 100%/100%. 8. Prior carfilzomib: 84.2%. 9. Prior pomalidomide: 84.2%. 10. Prior daratumumab: 94.7%.	IV CC-93269 on days 1, 8, 15, and 22 of cycles 1 to 3, on days 1 and 15 of cycles 4 to 6, and on day 1 of cycle 7 (28-day cycle, up to 2 tears)	1. ORR: <6 mg: No response. ≥ 6mg: 83.3% (10/12) (MRD-: 75%).	1. Gr 3-4 AE: 15 (78.9%) 2. Common AEs: Neutropenia (52.6%), anemia (42.1%), infections (26.3%), thrombocytopenia (21.1%). 3. CRS: 89.5% (n=17), including 11 Gr 1 and 2 Gr2. 4. One death due to CRS (≥ 6mg cohort).
Talquetamab (JNJ-64407564) GPRC5D x CD3	1. Phase 1 (MonumenTAL-1, NCT03399799). 2. RRMM, n=74. 405 μg/kg QW (n=30). 800 μg/kg Q2W (n = 44). 3. Prior lines of Tx: 6 (405)/5 (800). 4. Triple exposed: 100% (405)/98% (800). Triple refractory: 77% (405)/75% (800).	1. 405 μg/kg SC QW or 800 μg/kg SC Q2W	ORR (405/800) 1. 70% (21/30)/64% (28/44) 2. ≥ VGPR:57%/52%	AEs (405/800) 1. Cytopenia: 67%/36% (Gr 3/4: 53%/23%). 2. Infections: 47%/34% (Gr 3/4: 7%/9%). 3. CRS: 77%/80% (Gr 3: 3%/0%) 4. Skin-related and nail disorder: 83%/75% (skin exfoliation: 37%/39%, all Gr 1 and 2). 5. Dysgeusia: 63%/57%.
RG6234 (RO7425781) GPRC5D x CD3	1. Phase 1 (NCT04557150). 2. RRMM, n= 41 (IV, 0.006mg to 10mg) 3. Median age:63 4. Prior lines of Tx: 5 (2-15) 5. HR-cytogenetics: 58% (17/29) 6. Triple/Penta refractory: 67%/36% 7. Prior BCMA-direct Tx: 14.6% (6/41)	1. Weekly step-up doses followed by a q2w regimen at a peak ‘target dose’ for up to 1 year.	Efficacy evaluable, n=34 1.ORR: 68% (50% ≥ VGPR) 2. Median time to first response: 1.3 months.	1. CRS: 85.4% (Gr1: 56.1%, Gr2: 24.4%, Gr3: 2.4%). 2. CNS toxicity: 7.3% 3. Gr 3/4: Thrombocytopenia (19.5%), anemia (12.2%), neutropenia (9.8%). 4. Infection: 46.3%, one died of an E. coli sepsis (not related to RG6234). 5. Skin-related AEs: 66% (Gr3: 7.3%), dysgeusia/ageusia (36.6%, all Gr1/2), dry mouth (36.6%, all Gr1/2), dysphagia (17.1%, all Gr1/2), and nail changes (12.2%, all Gr1/2).
Cevostamab (BFCR4350A) FcRH5 x CD3	1. Phase 1 (NCT03275103). 2. RRMM, n=160. 3. Median age: 64 years (range: 33-82). 4. Prior lines of Tx: 6 (2-18). 5. Triple refractory: 85% 6. ≥ 1 prior CAR-T: 17.5% 7. ≥ 1 prior BiAb: 8.1% 8.≥ 1 prior ADC: 16.9% 9.≥ 1 anti-BCMA: 33.8%	1. Single set-up cohort (SS): set-up dose (0, 05-3.6mg) on cycle (C) 1 day (D) 1, target dose (0.15-198 mg) on C1D8. 2. Double set-up cohort (DS): set-up dose on C1D1 (0.3-1.2mg) and C1D8 (3.6mg), target dose (60-160mg) on C1D15. 3. IV cevostamab was administered on a 21-day cycle, up to a total of 17 cycles.	1. Responses (+) at the 20-198mg target dose level. 2. Median time to response: 29 days 3.ORR: 160mg level: 54.5% (24/44). 90mg level: 36.7% (22/60). 4. ORR (> 90mg) Prior CAR-T: 44.4% (4/9). Prior BiAb: 33.3 (3/9). Prior ADC: 50% (7/14). Prior anti-BCMA Tx: 36.4% (8/22).	1. ≥1 TEAE: 99.4%. 2. CRS: 80% (128/160), only 2 Gr 3. 3. Most CRS (83.4%) resolved within 2 days. 4. ICANS: 13.1% 5. Any SAE: 55.6% (Tx related: 25%) 6. Any Gr 5 AE: 15% (Tx related: 0.6%).

ADC, antibody-drug conjugate; AE, adverse event; CAR-T, chimeric antigen receptor T cell; CNS, central nervous system; CR, complete remission; CRS, cytokine releasing syndrome; DLT, dose-limiting toxicity; EMD, extramedullary disease; ESC, dose escalation; EXP, dose expansion; Gr, grade; HR, high-risk; HSCT, hematopoietic stem cell transplantation; ICANS, immune effector cell-associated neurotoxicity syndrome; IMiDs, immunomodulatory drugs; IV, intravenous; MoAb, monoclonal antibody; MRD, minimal residual disease; NR, non-reached; ORR, overall (objective) response rate; PI, proteasome inhibitor; PR, partial response; RP2D, Recommended Phase 2 Dose; SAE, serious AE; SC, subcutaneous; TEAE, treatment emerged AE; Tx, treatment; URI, upper respiratory tract infection; VGPR, very good partial response.

In addition, BiAb/BiTEs may have a lower risk of severe immune cell activation–related AEs, like CRS or neurotoxicity, and a lower chance of target antigen loss than CAR-T cell therapy (93). Importantly, based on the findings in clinical trials, these BiAb/BiTE molecules continue to achieve treatment responses better than those of ADC and close to those of CAR-T cell therapy, with an adequate safety profile, if an adequate therapeutic dose is defined (94). The comparison between T-cell directing BiAb/BiTE and CAR-T cell therapy is listed in **Table 2**.

**Table 2:** T-cell-redirecting BiAb/BiTE vs CAR-T cell therapy in MM.

	BiAb/BiTE	CAR-T
Structure	BiAb: Engineered artificial antibodies to recognize two epitopes of an antigen or two antigens. BiTE: A recombinant protein composed of two linked scFvs, with one targeting CD3 and the other one targeting MM antigen.	A synthetic receptor composed of a target antigen-binding domain (scFv), a hinge region, a transmembrane domain, and intracellular signaling domains.
Immune synapse	Typical	Atypical
Effector cells	CD4 and CD8 cells	CD4 and CD8 cells
Availability	Off the shelf	1. Maybe > 2 weeks for manufacture. 2. Rapid manufacturing process is under development.
Manufacturing failure	Not applicable	Around 10%
Administration	1. No conditioning treatment. 2. Pretreatment: steroid. 3. Repeat dosing.	1. Conditioning treatment (+). 2. Pretreatment: anti-histamine, acetaminophen. 3. One-time infusion.
The treatment response rate in RRMM	1. Generally lower. 2. It may be similar to CAR-T therapy in patients treated with top doses or at the RP2D.	Generally higher
Target antigen loss	Lower risk	Higher risk
CRS risk (≥ Gr 3)	1. Generally lower. 2. Increase with a higher dose.	Generally higher.
Neurotoxicity (≥ Gr 3)	Lower	Higher
Financial burden	Expensive	Expensive
FDA approval	Talquetamab (2022).	Idecabtagene vicleucel (2021) Ciltacabtagene autoleucel (2022).
EMA approval	Teclistamab (2022).	Idecabtagene vicleucel (2021).

Gr, grade; scFV, single-chain fragment variable

## Perspectives

Most of the current BiAb/BiTEs target BCMA, yet BCMA-based immunotherapy is a highly competitive and crowded field. Moreover, the COVID-19 pandemic also significantly affected the enrollment of clinical trials (95). These factors may negatively impact the development of certain anti-BCMA agents. Exploring other MM-specific antigens could be a potential solution to overcome BCMA-treatment failure and would serve as a strategy for developers to move into less crowded fields.

To reduce the risk of low antigen expression or antigen loss–related relapse, combining bispecific molecules that target different tumor antigens would be a logical solution. For example, talquetamab (anti-BCMA) and teclistamab (anti-GPRC5D) are under evaluation in RRMM patients (NCT04586426). Moreover, pharmacologic modulation to increase antigen expression is also an attractive strategy for clinical investigation. The utilization of γ-secretase inhibitor at low doses to increase BCMA expression was evaluated in combination with anti-BCMA agents (**Table 3**).

**Table 3:** Summary of combination strategies of bispecific antibodies in clinical trials.

Strategy 1: Combination with other anti-myeloma agents	
Rationale: Potential synergistic effect, reduced tumor burden.	
Trial No.	Agents
NCT03287908 (Phase 1)	Pavurutamab (AMG 701) monotherapy Pavurutamab + pomalidomide Pavurutamab + pomalidomide + dexamethasone
NCT04108195 TriMM-2 (Phase 1)	Talquetamab + daratumumab Teclistamab + daratumumab Then ± pomalidomide
NCT05090566 MagnetisMM-4 (Phase 2)	Sub-study B Elranatamab + lenalidomide + dexamethasone
NCT05020236 MagnetisMM-5 (Phase 3)	Elranatamab vs daratumumab+ pomalidomide+ dexamethasone Elranatamab + daratumumab vs daratumumab+ pomalidomide+ dexamethasone
NCT05137054 (Phase 1)	Linvoseltamab (REGN5458) + daratumumab + dexamethasone Linvoseltamab + carfilzomib + dexamethasone Linvoseltamab + lenalidomide + dexamethasone Linvoseltamab + bortezomib + dexamethasone
**Strategy 2: Combination of 2 bispecific molecules targeting various MM antigens**	
**Rationale: To reduce the risk of antigen loss related disease relapse.**	
NCT04586426 (Phase 1)	Part 2: Dose expansion cohort Talquetamab + teclistamab Talquetamab + teclistamab + daratumumab
**Strategy 3: Combined agent which enhances expression of target antigen**	
**Rationale: Enhanced antigen expression increased anti-MM activity of bispecific molecules**	
NCT04722146 (Phase 1)	Talquetamab + nirogacestat
NCT05090566 MagnetisMM-4 (Phase 2)	Sub-study A Elranatamab + nirogacestat

Since BiAb/BiTEs have shown encouraging treatment outcomes in RRMM patients, some studies exploring their role in earlier lines of treatment are ongoing. In fact, the anti-CD19 BiTE blinatumomab increases the MRD negativity rate after chemotherapy in patients with acute lymphoblastic leukemia. A phase 3 trial evaluating elranatamab vs lenalidomide in newly diagnosed MM after autologous transplantation has been initiated (MagnetisMM-7, NCT05317416). For the BiAb teclistamab, there are 2 clinical trials are ongoing. The first one is a phase 2 trial aiming to investigate the role of the teclistamab in post-transplantation maintenance therapy (Master-2, NCT05231629). The other one is a phase 3 trial of teclistamab in combination with lenalidomide versus lenalidomide alone as the maintenance therapy in patients with newly diagnosed MM following autologous stem cell transplantation (MajesTEC-4, NCT05243797).

Recently, a study demonstrated that utilization of treatment-free intervals (between treatment cycles) may transcriptionally reprogram and functionally reinvigorate T cells to lower the possibility of T cell exhaustion, an important factor related to suboptimal treatment results or treatment failure in cancer immunotherapy. The findings provide important reference for further protocol optimization of T-cell-recruiting therapies (96).

## Conclusion

Despite significant advances in MM therapy, the existing anti-MM agents are still largely ineffective for patients with high-risk and RR MM. Impressively, novel targeted immunotherapies, especially BiAb/BiTEs, have emerged as promising monotherapies in heavily pretreated RRMM. With more clinical studies showing favorable clinical efficacies, more bispecific molecules will enter clinical development and be approved for myeloma treatment. Moreover, we can expect the introduction of more optimized treatment protocols incorporating these cutting-edge immunotherapeutic agents to further restore overall anti-MM immunity and improve the quality of life.

## Author contributions

S-FC and Y-TT outlined and wrote the manuscript. S-FC, T-JY, and Y-TT performed literature research. S-FC, T-JY, and Y-TT created the figure. S-FC, KA, and Y-TT critically reviewed, edited, and revised the manuscript. All authors read and approved the final manuscript.

## Funding

This work is supported in part by NIH/NCI grants SPORE-P50CA100707, R01CA207237, and P01CA155258; by the Adelson Medical Research Foundation and the Paula and Rodger Riney Foundation; by a grant from Kaohsiung Medical University Hospital (KMUH109-9M22 and SA11002) and the Taiwan Ministry of Science and Technology (MOST 110-2628-B-037-009, 111-2314-B-037-050-MY2). These funders had no role in the study design, data collection, data analysis, decision to publish, or preparation of the manuscript.

## Acknowledgments

We thank all laboratory and clinical research teams at the LeBow Institute for Myeloma Therapeutics and the Jerome Lipper Multiple Myeloma Center of the Dana-Farber Cancer Institute for their continuous encouragement, help, and support. We also thank Dr. Christina Usher for critical review and helpful input.

## Conflict of interest

Author KA serves on advisory boards to Pfizer, Amgen, AstraZeneca, Janssen, Precision Biosciences, Window, and Starton, and is a scientific founder of OncoPep, C4 Therapeutics, Raqia, and NextRNA.

The remaining authors declare that the research was conducted in the absence of any commercial or financial relationships that could be construed as a potential conflict of interest.

## Publisher’s note

All claims expressed in this article are solely those of the authors and do not necessarily represent those of their affiliated organizations, or those of the publisher, the editors and the reviewers. Any product that may be evaluated in this article, or claim that may be made by its manufacturer, is not guaranteed or endorsed by the publisher.
