# Supplementary material for: Bispecific antibodies in multiple myeloma treatment: A journey in progress
Source: Front Oncol. 2022 Oct 18;12:1032775. doi: 10.3389/fonc.2022.1032775 (PMC9623099; doi:10.3389/fonc.2022.1032775)
Supplement: Supplementary file 1 [file Table_1.pdf]

Supplementary Table 1. Summary of clinical trials of BiTE/BiAb (Out of pipeline).

Agents (target)	Trial and participant data	Protocol	Efficacy	Safety
Pacanalotamab<br>(AMG 420,<br>BI 836909)<br>BCMA x CD3	1. Phase 1 trial (NCT02514239)<br>2. RRMM, n= 42<br>3. Median age: 65 (range: 39-79).<br>4. Prior lines of Tx: 5<br>5. HR cytogenetics: 33%<br>6. Prior daratumumab: 29%<br>7. Prior elotuzumab: 10%<br>8. Prior auto-HSCT: 86%	1. Dose escalation study.<br>2. Six-week cycles of AMG 420 up to 10 cycles (4 weeks continuous IV infusion + 2 weeks off).<br>3. Single-patient cohorts [0.2–1.6 µg/day (d)] followed by cohorts of 3–6 patients (3.2–800 µg/d).	1. ORR: 31% (13/42).<br>2. Responses (+) at 6.5 µg/d.<br>3. Median time to response: 1.4 months.<br>4. ORR at 400 µg/d: 70% (7/10), including 5 MRD-negative CRs, 1 VGPR, and 1 PR.	1. SAE: 20 (48%), including infection (n= 14) and polyneuropathy (n= 2).<br>2. CRS: 16, including 2 Gr 2 and 1 Gr 3.<br>3. No Gr≥ 3 CNS toxicities.<br>4. DLT (+): 2 patients (2/3, at 800µg/d)
Pavurutamab<br>(AMG 701)<br>BCMA x CD3	1. Phase 1 (NCT03287908)<br>2. RRMM, n= 75<br>3. Median age: 63 years<br>4. Prior lines of Tx: 6 (1-25)<br>5. EMD: 27%<br>6. Prior HSCT: 83%<br>7. Prior anti-CD38 MoAb: 93%<br>8. Triple refractory: 68%	1. AMG 701 IV infusions: weekly in 4-week cycles until PD.<br>2. Target doses (mg): 0.14, 0.4, 0.8, 1.2, 1.6. 3.0, 4.5, 6.5, 9, 12.	1. ORR: 36% (16/45) at doses of 3-12 mg.<br>2. ORR: 83% (5/6, 3 PRs, 2 VGPRs) (earlier dose escalation with 9 mg).<br>3. Median time to response: 1.0 month.<br>4. MRD in 4 CR patients: all negative (4/4).	1. Hematological AEs: anemia (43%), neutropenia (23%), and thrombocytopenia (20%).<br>2. Non-hematological AEs: CRS (61%), diarrhea (31%), fatigue (25%), and fever (25%).<br>3. All Gr 3 CRS (n= 5, 7%) were assessed as DLTs.<br>4. SAE (n= 29, 39%): infections (n= 13), CRS (n= 7), and asymptomatic pancreatic enzyme rise (n= 2).<br>5. Neurotoxicity: 6 (4 CRS-related, all Gr 1/2)

AE, adverse event; CNS, central nervous system; CR, complete remission; CRS, cytokine releasing syndrome; DLT, dose-limiting toxicity; EMD, extramedullary disease; Gr, grade; HR, high-risk; HSCT, hematopoietic stem cell transplantation; IV, intravenous; MoAb, monoclonal antibody; MRD, minimal residual disease; ORR, overall (objective) response rate; PR, partial response; SAE, serious AE; Tx, treatment; VGPR, very good partial response.
